# Catalytic Activity of Polynuclear vs. Dinuclear Aroylhydrazone Cu(II) Complexes in Microwave-Assisted Oxidation of Neat Aliphatic and Aromatic Hydrocarbons

**DOI:** 10.3390/molecules24010047

**Published:** 2018-12-23

**Authors:** Manas Sutradhar, Tannistha Roy Barman, Armando J. L. Pombeiro, Luísa M.D.R.S. Martins

**Affiliations:** Centro de Química Estrutural, Instituto Superior Técnico, Universidade de Lisboa, Av. Rovisco Pais, 1049-001 Lisboa, Portugal; tannisthachem@gmail.com (T.R.B.); pombeiro@tecnico.ulisboa.pt (A.J.L.P.)

**Keywords:** Cu(II) complexes, aroylhydrazone, X-ray structure, microwave, oxidation, alkane, hydrocarbon, cyclohexane, toluene

## Abstract

One-dimensional (1D) polynuclear Cu(II) complex (**1**) derived from (5-bromo-2-hydroxybenzylidene)-2-hydroxybenzohydrazide (H_2_L) is synthesized and characterized by elemental analysis, IR spectroscopy, ESI-MS, and single crystal X-ray crystallography. Its catalytic performance towards the solvent-free microwave-assisted peroxidative oxidation of aliphatic and aromatic hydrocarbons under mild conditions is compared with that of dinuclear Cu(II) complexes (**2** and **3**) of the same ligand, previously reported as antiproliferative agents. Polymer **1** exhibits the highest activity, either for the oxidation of cyclohexane (leading to overall yields, based on the alkane, of up to 39% of cyclohexanol and cyclohexanone) or towards the oxidation of toluene (selectively affording benzaldehyde up to a 44% yield), after 2 or 2.5 h of irradiation at 80 or 50 °C, respectively.

## 1. Introduction

The search for efficient routes for the mild oxidative functionalization of hydrocarbons, in particular of inert alkanes, to valuable commodities using suitable catalysts is a demanding research area of high industrial significance [1,2,3]. In fact, although alkanes are relatively cheap and highly abundant raw materials for the direct synthesis of added-value functionalized products, due to their chemical inertness, the design of active and selective catalysts for the atom-efficient and selective oxidation of the low reactive C–H bond continues to be a real challenge [4,5,6,7].

Nature uses earth-abundant first-row transition metals for small molecules activation, as well as selective functionalization of particular inert substrates. However, attempts to model metalloenzyme active sites often suffer from low efficiency for the desired reactions. 

In general, the rationale to design synthetic bio-inspired catalyst precursors for functionalizing the non-activated C–H bonds of hydrocarbons is based on metallic complexes, where the metal center can undergo reversible redox processes. The most tested transition metal complexes as catalysts for such oxidation reactions bear tridentate pincer [8,9,10,11] or scorpionate [4,5,12] ligands, or are coordination polymers [13], metal-based pocket structures (e.g., cyclodextrins or calixarenes) [14], or metal–organic frameworks [15]. 

Different copper(II) complexes [4,5,16,17,18,19,20,21,22,23,24], including two tautomeric aroylhydrazone Cu(II) complexes [20,23], were found to exhibit high catalytic activity under mild conditions for the peroxidative oxidation of cycloalkanes to the corresponding alkyl hydroperoxides, alcohols, and ketones. Therefore, the use of Cu(II) catalysts in oxidation reactions can be a wise choice due to their abundance, low cost, and non-toxic properties [25].

The partial oxidation of toluene usually gives rise to several products, among which are benzaldehyde, benzyl alcohol, benzyl acetate, benzoic acid, or cresols [26]. Therefore, a more selective process is needed, namely towards the production of benzaldehyde due to its importance as a commodity.

The use of microwave irradiation (MW) to assist such aliphatic and aromatic hydrocarbon oxidation reactions can contribute to the development of sustainable processes, with advantages over conventional heating methods thanks to its simplicity, accelerating power, and energy-saving technique [27,28,29,30,31].

Herein, we report the synthesis and characterization of a new one-dimensional (1D) Cu(II) aroylhydrazone polymer derived from (5-bromo-2-hydroxybenzylidene)-2-hydroxybenzohydrazide (H_2_L) aroylhydrazone ligand (**1**). The catalytic performance of this polymeric Cu(II) complex towards the MW-assisted peroxidative oxidation of aliphatic and aromatic hydrocarbons is compared with the ability of two biologically active [32] dinuclear Cu(II) complexes bearing mixed ligands [CuL(Pz)]_2_ (**2**) or [CuL(Py)]_2_ (**3**) (Pz = pyrazole; Py = pyridine) derived from the same ligand. 

## 2. Results and Discussion

### 2.1. Synthesis and Characterization

**Scheme 1:**
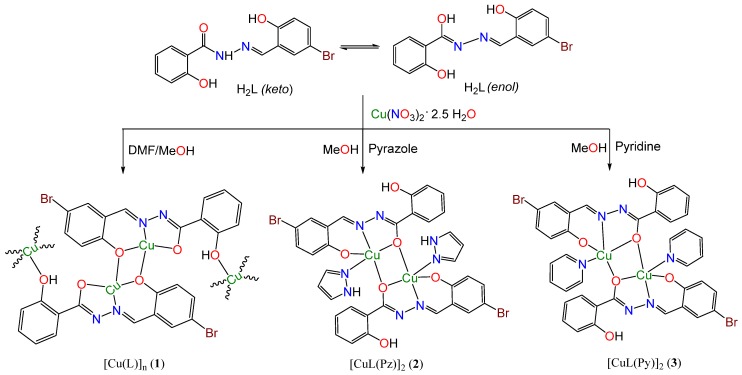
Synthesis of **1**–**3**.

The aroylhydrazone Schiff base (5-bromo-2-hydroxybenzylidene)-2-hydroxybenzohydrazide (H_2_L) [32] was used to synthesize a new 1D polynuclear Cu(II) complex **1** (Scheme 1). The dinuclear complexes **2** and **3** of the same ligand (Scheme 1), reported earlier for their biologic activity [32], were also prepared to assess their catalytic activity towards the solvent-free MW-assisted peroxidative oxidation of aliphatic and aromatic hydrocarbons under mild conditions, and this was compared with the performance of the new polymer **1** under the same conditions. 

It was previously observed that the reaction of the aroylhydrazone with a Cu(II) salt of a base of a strong acid yielded an aroylhydrazone-Cu(II) complex in the keto form, whereas a Cu(II) salt of a base of a weak acid produced a Cu(II) complex with the enol form [20,23]. In this work, the reaction of H_2_L with Cu(NO_3_)_2_·2.5H_2_O in DMF/methanol medium afforded the 1D aroylhdyrazone-Cu(II) polymer [Cu(L)]_n_ (**1**) with the ligand in the enol form (Scheme 1). The reaction of Cu(NO_3_)_2_·2.5H_2_O with the aroylhydrazone in the presence of the *N*-donor Lewis base pyrazole (Pz) or pyridine (Py) led to the mixed ligand complexes [CuL(Pz)]_2_ (**2**) or [CuL(Py)]_2_ (**3**), respectively (Scheme 1) [32], via the enolization and deprotonation of the aroylhydrazone. 

The characterization of complex **1** was carried out by elemental analysis, IR spectroscopy, ESI-MS, and single crystal X-ray diffraction techniques. 

The IR spectrum of **1** displays the C=N and (C–O) enolic stretching frequencies at 1608 and 1252 cm^−1^, respectively, apart from other characteristic bands of the ligand [32]: 3454 ν (OH) and 1158 ν (N–N). The *m*/*z* value of **1** indicates the decomposition of the polynuclear species to a mononuclear one in solution, which was also observed in the case of **2** and **3**. 

**Figure 1:**
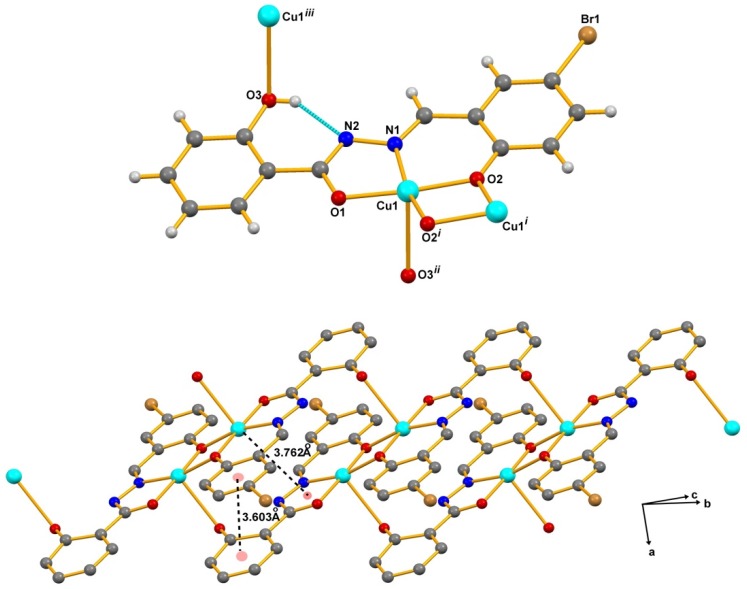
Structural representation of **1** with partial atom labeling scheme (**top**). H-bond interactions are represented by the dashed light blue color. Symmetry codes to generate equivalent atoms are as follows: (i) 2 − *x*, 1 − *y*, 1 − *z*; (ii) *x*, 1 + *y*, *z*; (iii) *x*, −1 + *y*, *z*. The infinite 1D chain of **1** runs along the crystallographic a axis (**bottom**).

The X-ray crystal of **1** was obtained upon slow evaporation from a methanol-DMF mixture at room temperature. The crystallographic data for **1** (CCDC 1881482, see supplementary materials) are summarized in Table 1 and selected dimensions are presented in Table 2. The molecular structure of **1** is displayed in Figure 1. 

The ligand (L^2−^) in **1** is almost planar; two ligands in sum behaves as a hexadentate chelator and acts as a NO_2_ donor for each metal cation in **1**, as observed in other cases [33,34]. The copper cations in **1** exhibit distorted square pyramidal geometries (τ5 of ca. 0.017) [35,36]. The structure of **1** exists in a 1D coordination polymer (Figure 1) with the aroylhydrazone ligand binding the metal cations in the dianionic (L^2−^) form and acting as a bridging chelate ligand by means of the O_phenolate_-, the O_enolate_-, and the N_imine_ atoms. The metal basal planes in **1** are involved in Cu_2_(O_phenolate_)_2_ cores and are complemented by the O_enolate_- and the N_imine_ atoms; the apical positions are engaged with the O_phenolic_ atoms from symmetry-related ligands. The metal cation is deviated 0.108 Å from the plane defined by the basal binding atoms, which are involved (in addition to the Cu_2_(O_phenolate_)_2_ core four-membered rings) in five- and six-membered metallacycles, viz. CuOCN_2_ and CuOC_3_N, respectively. 

The compound is stabilized by hydrogen bond interactions. The π···π stacking interactions are observed in **1,** which contribute to the reinforcement of the 1D polymeric structure. Such interactions are not only found between the phenolic and the phenolate rings, whose centroids are 3.603 Å apart, but also involve the five-membered metallacycles with centroids distant from a vicinal copper cation by 3.762 Å [37].

### 2.2. Microwave-Assisted Peroxidative Oxidation of Cyclohexane and Toluene

**Scheme 2:**
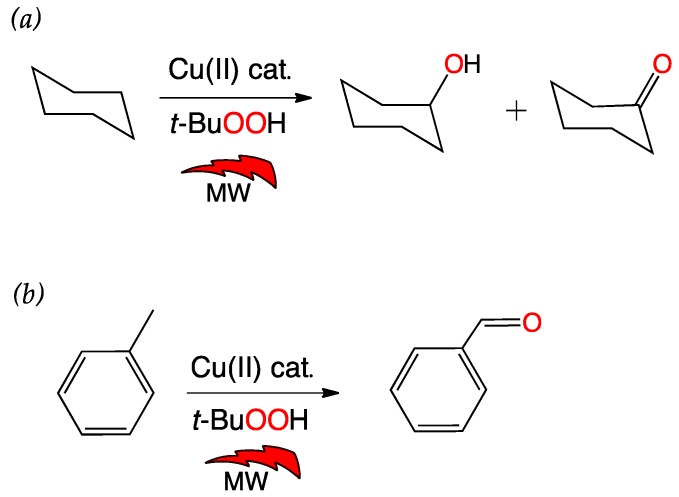
Microwave-assisted oxidation of cyclohexane (**a**) or toluene (**b**) catalyzed by complexes **1**–**3**.

Following our attempts [17,18,19,20,21,22,23] to use Cu(II) complexes to catalyze the oxidation of cyclohexane with aqueous hydrogen peroxide, usually in MeCN and at room temperature, herein compounds **1**–**3** were tested as homogeneous catalysts for the microwave-assisted neat oxidation (without added solvent) of cyclohexane or toluene with aq. tert-butyl hydroperoxide (*t*-BuOOH, TBHP) to the corresponding oxygenates (Scheme 2). Cyclohexane and toluene were chosen as model aliphatic and aromatic hydrocarbon substrates due to their significance as industrial substrates.

Cyclohexane was successfully converted with cyclohexanol and cyclohexanone as the only products (Scheme 2a). No other products were detected by GC-MS analysis under the assayed conditions, thus revealing a very selective oxidation system. In addition, very good yields of up to 39% for **1** (ca. four times higher than those reported for the industrial aerobic process to guarantee a maximum selectivity) [1] of KA oil (ketone-alcohol oil i.e., cyclohexanol and cyclohexanone mixture) were obtained after 2 h of MW irradiation at 80 °C in the absence of any promotor or co-catalyst. Dinuclear complexes were slightly less effective in forming KA oil than **1**, leading to total yields of 26% and 31% for **2** and **3**, respectively, under the same reaction conditions. The presence of a Lewis base can decrease the Lewis acidic character of the Cu(II) center in **2** and **3**. Thus, the absence of a coordinated Lewis base in **1** appears to promote the catalytic activity of the Cu(II) center in **1.**

**Figure 2:**
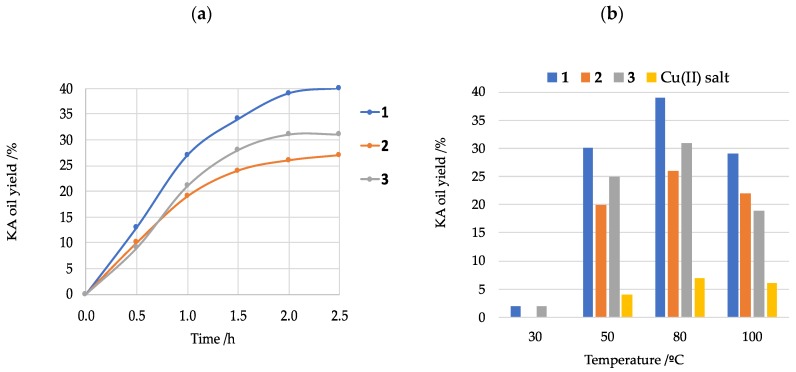
Effect of reaction time (**a**, T = 80 °C) and temperature (**b**, 2 h) on the yields of ketone-alcohol (KA) oil obtained by the microwave (MW)-assisted oxidation of cyclohexane catalyzed by complexes **1**–**3**.

The best catalytic results for complexes **1**–**3** were achieved for reactions carried out at 80 °C after 2 h of microwave irradiation (see Figure 2). After this time under the above conditions, the yield enhancement was not significant (Figure 2a). The attempt to perform such reactions at lower or higher temperatures (30–100 °C range) resulted in marked drops in the yield (Figure 2b). The relevance of the *N*, *O*-donor ligands was shown by comparison of the catalytic activity of Cu(NO_3_)_2_, which, at optimized conditions, led to a 7% maximum yield of KA oil (Figure 2b).

In order to establish the nature of the cyclohexane oxidation mechanism, experiments to detect the formation of cyclohexyl hydroperoxide as a primary product, following a method proposed by Shul’pin [38], were performed. Accordingly, the detected alcohol amount significantly increased after the addition of triphenylphosphine to the reaction mixture, as a result of the reduction of cyclohexyl hydroperoxide to cyclohexanol. A concomitant decrease on the amount of cyclohexanone was observed in these conditions, suggesting the formation of cyclohexyl hydroperoxide as the primary product of this reaction. Further experiments involving the addition of oxygen- or carbon-radical traps (Ph_2_NH or CBrCl_3_, respectively) to the reaction mixture led to a significant yield drop of over 90%, when compared to the reaction performed under the same conditions but in the absence of such a radical trap. Thus, oxidation of cyclohexane catalyzed by complexes **1**–**3** should proceed through a radical mechanism involving both carbon- and oxygen-centered radicals, as depicted in the following reactions (1)–(9). Firstly, the Cu-catalyzed decomposition of TBHP occurs, leading to *t*-BuOO˙ and *t*-BuO˙ radicals upon oxidation by Cu(II) or reduction by Cu(I) species, according to reactions (1) and (2), respectively. Then, H-abstraction from CyH by *t-*BuO˙ leads to the formation of cyclohexyl radical (Cy˙) (reaction (3)). CyOO˙ is formed by the reaction of Cy˙ with dioxygen (reaction (4)), and CyOOH can then be formed upon H-abstraction from TBHP by CyOO˙ (reaction (5)). The Cu-assisted decomposition of CyOOH to CyOO˙ and CyO˙ (reactions (6) and (7)) leads to the formation of the desired products: cyclohexanol (CyOH) and cyclohexanone (Cy_-H_=O), according to reactions (8) and (9).

[Cu^II^] + *t*-BuO_2_H → *t*-BuOO˙ + H^+^ + [Cu^I^], (1)

[Cu^I^] + *t*-BuO_2_H → *t*-BuO˙ + [Cu^II^] + HO^−^, (2)

*t*-BuO˙ + CyH → *t*-BuOH + Cy˙, (3)

Cy˙+ O_2_ → CyOO˙, (4)

CyOO˙ + *t*-BuO_2_H → CyOOH + *t*-BuOO˙, (5)

CyOOH + [Cu^I^] → CyO˙ + HO^−^ + [Cu^II^], (6)

CyOOH + [Cu^II^] → CyOO˙ + H^+^ + [Cu^I^], (7)

CyO˙ + CyH → CyOH + Cy˙,(8)

2CyOO˙ → CyOH + Cy_-H_=O + O_2_, (9)

In addition, in order to try to detect which radical (*t*-BuO˙ or *t*-BuOO˙) is the active oxidant, cyclohexane oxidation was performed with di-tert-butylperoxide, *t*-BuOO*t*-Bu (*t*-BuOO· could not be formed in its homolytic cleavage), for the most active catalyst **1** under the above optimized reaction conditions. A lower yield (27%) of KA oil was obtained, indicating the relevant role of *t*-BuOO˙ radical in the above mechanism.

The oxidation of toluene proceeded with the oxygenation of the methyl group, yielding benzaldehyde (Scheme 2b) as the major product (selectivity up to 99% relative to benzyl alcohol), while benzyl alcohol and benzoic acid (the latter detected only for high concentrations of catalyst) were very minor products (<1%). Moreover, no oxidation of the aromatic ring was observed under the assayed conditions. A maximum benzaldehyde yield of 44% was achieved after 2.5 h of MW irradiation, at 50 °C, catalyzed by **1**, in the absence of any promotor or co-catalyst. As observed for the oxidation of cyclohexane, dinuclear complexes were also less effective in converting toluene than **1**, leading to total yields of 30 and 38% for **2** and **3**, respectively, under the same reaction conditions.

**Figure 3:**
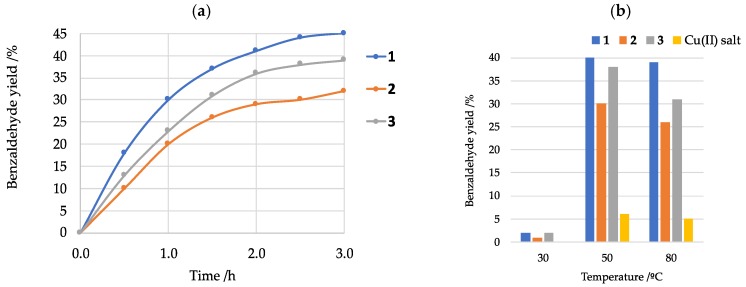
Effect of reaction time (**a**, T = 50 °C) and temperature (**b**, 2.5 h) on the yields of benzaldehyde obtained by the MW-assisted oxidation of toluene catalyzed by complexes **1**–**3**.

The best catalytic results for complexes **1**–**3** were achieved for reactions carried out at 50 °C after 2.5 h of microwave irradiation (see Figure 3). After this time under the above conditions, the yield enhancement was not significant (Figure 3a). The attempt to perform such reactions at lower or higher temperatures (30–80 °C range) resulted in marked drops in the yield (Figure 3b). The relevance of the *N*, *O*-donor ligands for the catalytic activity of the copper center was shown by comparison of the catalytic activity of Cu(NO_3_)_2_, which, at optimized conditions, led to a 6% maximum yield of benzaldehyde (Figure 3b).

Experiments in the presence of 2,2,6,6-tetramethylpiperidyl-1-oxyl (TEMPO) led to a significant yield drop (over 80%). Thus, the oxidation of toluene catalyzed by complexes **1**–**3** should proceed through a radical mechanism, as depicted in the following reactions (1), (2), and (10)–(12). Firstly, the Cu-catalyzed decomposition of TBHP occurs, leading to *t*-BuOO˙ and *t*-BuO˙ radicals upon oxidation by Cu(II) or reduction by Cu(I) species, according to reactions (1) and (2), respectively. Then, *t*-BuO˙ abstracts an H atom from toluene, affording the benzyl radical C_6_H_5_CH_2_˙(reaction (10)), which, upon reaction with O_2_, forms the benzyl peroxyl radical C_6_H_5_CH_2_OO˙(reaction (11)). The dismutation of C_6_H_5_CH_2_OO˙ leads to benzyl alcohol and benzaldehyde (reaction (12)). In addition, benzaldehyde can be formed upon the further Cu-promoted oxidation of benzyl alcohol with TBHP.

*t*-BuO˙ + C_6_H_5_CH_3_ → t-BuOH + C_6_H_5_CH_2_˙, (10)

C_6_H_5_CH_2_˙+ O_2_ → C_6_H_5_CH_2_OO˙, (11)

2 C_6_H_5_CH_2_OO˙ → C_6_H_5_CH_2_OH + C_6_H_5_CHO + O_2_, (12)

The catalytic systems based on the Cu(II) complexes **1**–**3** were found to be very effective in the microwave-assisted solvent- and promotor-free peroxidative oxidation of cyclohexane and toluene, despite the low power of MW used. For example, under the same reaction conditions, but replacing MW by an oil-bath as a heating source, only 7% of benzaldehyde was formed after a 2.5-h reaction at 50 °C in the presence of **1**. Moreover, the extension of the reaction time to 24 h under the above conditions afforded a still low (24%) benzaldehyde yield. The successful use, in these catalytic oxidations, of microwave heating and of solvent- and promotor-free conditions is of environmental significance and contributes to the enhancement of the sustainability of both catalytic processes.

## 3. Materials and Methods

### 3.1. General Materials and Equipment

The synthetic part of this study was performed in air. Commercially available reagents and solvents were used as received, without further purification or drying. Cu(NO_3_)_2_·2.5H_2_O was used as the metal source for the synthesis of complexes **1**–**3**.

C, H, and N elemental analyses were carried out by the Microanalytical Service of the Instituto Superior Técnico, Lisboa, Portugal. 

Infrared spectra (4000–400 cm^−1^) were recorded on a Bruker Vertex 70 (Bruker Corporation, Ettlingen, Germany) instrument in KBr pellets; wavenumbers are in cm^−1^. 

The ^1^H-NMR spectra were recorded at room temperature on a Bruker Avance II + 400.13 MHz (UltraShieldTM Magnet, Rheinstetten, Germany) spectrometer. Tetramethylsilane was used as the internal reference and the chemical shifts are reported in ppm. 

Mass spectra were run in a Varian 500-MS LC Ion Trap Mass Spectrometer (Agilent Technologies, Amstelveen, The Netherlands) equipped with an electrospray (ESI) ion source. For electrospray ionization, the drying gas and flow rate were optimized according to the particular sample with a nebulizer pressure of 35 psi. Scanning was performed from *m*/*z* 100 to 1200 in methanol solution. The compounds were observed in the positive mode (capillary voltage = 80–105 V). 

The microwave-assisted solvent-free peroxidative oxidations of cyclohexane and toluene were performed in G10 borosilicate glass tubes (10-mL capacity reaction tube with a 13-mm internal diameter) in a focused Anton Paar Monowave 300 reactor (Anton Paar GmbH, Graz, Austria) fitted with a rotational system and an IR temperature detector. 

Gas chromatographic (GC) measurements were carried out using a FISONS Instruments GC 8000 series gas chromatograph with a Flame Ionization Detector (FID) and a capillary column (DB-WAX, column length: 30 m; internal diameter: 0.32 mm) (Agilent Technologies, Santa Clara, CA, USA) and Jasco–Borwin v.1.50 software (Jasco, Tokyo, Japan) using helium as the carrier gas. The injection temperature was 240 °C. The initial temperature was maintained at 100 °C for 1 min, then raised 10 °C/min to 180 °C and held at this temperature for 1 min. 

Gas Chromatography-Mass Spectrometry (GC-MS) analyses were performed using a Perkin Elmer Clarus 600 C (Shelton, CT, USA) instrument (He as the carrier gas). The ionization voltage was 70 eV. Gas chromatography was conducted in the temperature-programming mode, using an SGE BPX5 column (30 m × 0.25 mm × 0.25 µm). 

All reaction products formed were identified by the comparison of their retention times, confirmed with those of commercially available samples. Reaction products mass spectra were compared to fragmentation patterns obtained from the NIST spectral library stored in the computer software of the mass spectrometer.

### 3.2. Syntheses of the Pro-Ligand H_2_L 

The aroylhydrazone Schiff base pro-ligand (5-bromo-2-hydroxybenzylidene)-2-hydroxybenzohydrazide (H_2_L) (Scheme 1) was prepared by a reported method [32] upon the condensation of the 2-hydroxybenzohydrazine with 5-bromo-2-hydroxybenzaldehyde. 

### 3.3. Synthesis of [Cu(L)]_n_ *(**1**)*

In brief, 0.335 g (1.0 mmol) of H_2_L was dissolved in 5 mL DMF, and 25 mL of a methanolic solution of Cu(NO_3_)_2_·2.5H_2_O (3.0 mmol) was added to it. The resultant mixture was stirred at 50 °C for 15 min; a dark green solution was obtained. The mixture was then filtered and the solvent was allowed to evaporate slowly. The use of methanol resulted in the precipitation of a solid powder instead of crystals; therefore, DMF was used to help the formation of good-quality (for X-ray diffraction analysis) crystals. After 1 day, single crystals suitable for X-ray diffraction were isolated, washed three times with cold methanol, and dried in open air.

Yield: 0.571 g (72%, with respect to Cu(II)). Anal. Calcd for (**1**) C_28_H_18_Br_2_Cu_2_N_4_O_6_: C, 42.39; H, 2.29; N, 7.06. Found: C, 42.34; H, 2.27; N, 7.02. IR (KBr; cm^−1^): 3454 ν(OH), 1608 ν(C=N), 1252 ν(C—O)enolic and 1158 ν(N–N). ESI-MS(+): *m*/*z* 415 [CuL(H_2_O) + H]^+^ (100%).

Complexes **2** and **3** were synthesized according to the protocol described in a previous work [32] by reacting Cu(NO_3_)_2_·2.5H_2_O and H_2_L in methanol in the presence of pyrazole (for **2**) or pyridine (for **3**), and they were characterized accordingly. 

### 3.4. X-Ray Measurements

Good-quality crystals suitable for the X-ray diffraction of **1** were isolated from a methanol-DMF mixture. A single crystal of **1** was immersed in cryo-oil, mounted in Nylon loops, and measured at a temperature of 150 K. Intensity data were collected using a Bruker APEX-II CCD diffractometer with graphite monochromated Mo-Kα (λ 0.71073) radiation. Data were collected using omega scans of 0.5° per frame and a full sphere of data was obtained. Cell parameters were retrieved using Bruker SMART [39] software and refined using Bruker SAINT [39] on all the observed reflections. Absorption corrections were applied using SADABS [39]. Structures were solved by direct methods by using SIR97 [40] and refined with SHELXL2014 [41]. Calculations were performed using WinGX v2014.1 [42]. All non-hydrogen atoms were refined anisotropically. Those H-atoms bonded to carbon were included in the model at geometrically calculated positions and refined using a riding model. U_iso_(H) was defined as 1.2 U_eq_ of the parent carbon atoms for phenyl and methylene residues. Least squares refinements with anisotropic thermal motion parameters for all the non-hydrogen atoms and isotropic for the remaining atoms were employed. 

### 3.5. Catalytic Studies 

The MW-assisted solvent-free peroxidative oxidations of cyclohexane and toluene were typically performed as follows: the desired amount of catalyst (5–20 μmol), substrate (cyclohexane or toluene), *t*-BuOOH (2 eq. vs. substrate, 70% aq. sol.) or *t*-BuOO*t*-Bu (2 eq. vs. substrate, 98%), and nitromethane (as an internal standard, 50 μL) were introduced into the Pyrex tube, closed, placed in the microwave reactor, and maintained under stirring and low power (5–10 W) irradiation at the desired temperature and for the desired reaction time. After the reaction, the obtained mixture was cooled to room temperature and centrifuged. A sample (4 µL) was taken from the organic phase and analyzed by GC upon the addition of an excess of triphenylphosphine to reduce cyclohexyl hydroperoxide to cyclohexanol, following a method developed by Shul’pin [38].

Blank experiments (without metal catalyst) were performed and no oxidation products were detected in the absence of the Cu(II) complexes or Cu salt.

## 4. Conclusions 

Polynuclear Cu(II) complex [Cu(L)]*_n_* (**1**) derived from (5-bromo-2-hydroxybenzylidene)-2-hydroxybenzohydrazide (H_2_L) shows high catalytic performance towards the solvent-free microwave-assisted peroxidative oxidation of aliphatic and aromatic hydrocarbons under mild conditions compared to the dinuclear Cu(II) complexes [CuL(Pz)]_2_ (**2**) or [CuL(Py)]_2_ (**3**), of the same ligand. Polymer **1** exhibits the highest activity, either for the oxidation of cyclohexane (leading to overall yields, based on the alkane, of up to 39% of cyclohexanol and cyclohexanone), or towards the oxidation of toluene (selectively affording benzaldehyde up to a 44% yield), after 2 or 2.5 h of microwave irradiation at 80 °C or 50 °C, respectively. This study shows the diversity of copper complexes towards the catalytic peroxidative oxidations of alkanes elicited by microwave irradiation under solvent-free conditions, which has a significant environmental implication.

## Figures and Tables

**Table 1:** Crystal data and structure refinement details for complex **1**.

	1
Empirical formula	C_14_H_9_BrCuN_2_O_3_
Formula weight	396.68
Crystal system	Monoclinic
Space group	*C*2/*c*
Temperature/K	150 (2)
*a*/Å	31.799 (2)
*b*/Å	6.3515 (4)
*c*/Å	14.2176 (10)
*α*/°	90
*β*/°	114.420 (4)
γ/°	90
*V* (Å^3^)	2614.6 (3)
*Z*	8
D_calc_ (g cm^−3^)	2.015
*μ*(Mo K*α*) (mm^−1^)	4.74
Rfls. collected/unique/observed	9952/2668/1906
*R*_int_	0.059
Final *R*1 *^a^*, *wR*2 *^b^* (*I* ≥ 2*σ*)	0.039, 0.074
Goodness-of-fit on *F*^2^	1.01

*^a^* R = Σ||*F_o_*| − |*F_c_*||/Σ|*F_o_*|; ^*b*^ wR(F^2^) = [Σw(|F_o_|^2^ − |F_c_|^2^)^2^/ Σw|F_o_|^4^]^½.^

**Table 2:** Selected bond distances (Å) and angles (°) in complex **1**.

N1—Cu1	1.914 (3)	O1—Cu1—O2	174.99 (11)
O1—Cu1	1.920 (2)	N1—Cu1—O2 ^i^	164.20 (13)
O2—Cu1	1.936 (2)	O1—Cu1—O2 ^i^	104.25 (11)
O2—Cu1 ^i^	1.977 (3)	O2—Cu1—O2 ^i^	80.16 (12)
Cu1—O2 ^i^	1.976 (3)	N1—Cu1—Cu1 ^i^	131.93 (10)
Cu1—Cu1 ^i^	2.9945 (9)	O1—Cu1—Cu1 ^i^	143.78 (8)
N1—Cu1—O1	82.22 (12)	O2—Cu1—Cu1 ^i^	40.58 (8)
N1—Cu1—O2	92.92 (12)	O2^i^—Cu1—Cu1 ^i^	39.58 (7)

Symmetry code: (i) −*x* + 2, −*y* + 1, −*z* + 1.

## Supplementary Materials

CCDC 1881482 for **1** contains the supplementary crystallographic data for this paper. This data can be obtained free of charge from The Cambridge Crystallographic Data Centre via www.ccdc.cam.ac.uk/data_request/cif. Supplementary data to this article can be found online.
