# Supplementary material for: Catalytic Activity of Polynuclear vs. Dinuclear Aroylhydrazone Cu(II) Complexes in Microwave-Assisted Oxidation of Neat Aliphatic and Aromatic Hydrocarbons
Source: Molecules. 2018 Dec 23;24(1):47. doi: 10.3390/molecules24010047 (PMC6337553; doi:10.3390/molecules24010047)

checkCIF/PLATON report

Structure factors have been supplied for datablock(s) shelx

THIS REPORT IS FOR GUIDANCE ONLY. IF USED AS PART OF A REVIEW PROCEDURE FOR PUBLICATION, IT SHOULD NOT REPLACE THE EXPERTISE OF AN EXPERIENCED CRYSTALLOGRAPHIC REFEREE.

No syntax errors found. CIF dictionary Interpreting this report

Datablock: shelx

```
Bond precision:  C-C = 0.0058 A          Wavelength=0.71073

Cell:            a=31.799(2)    b=6.3515(4)        c=14.2176(10)
                 alpha=90       beta=114.420(4)    gamma=90
Temperature:     150 K

                Calculated                Reported
Volume          2614.7(3)                 2614.6(3)
Space group     C 2/c                     C 2/c
Hall group      -C 2yc                    -C 2yc
Moiety formula  C14 H9 Br Cu N2 O3        C14 H9 Br Cu N2 O3
Sum formula     C14 H9 Br Cu N2 O3        C14 H9 Br Cu N2 O3
Mr              396.68                    396.68
Dx,g cm-3       2.015                     2.015
Z               8                         8
Mu (mm-1)       4.737                     4.737
F000            1560.0                    1560.0
F000’           1560.89
h,k,lmax        39,7,17                   39,7,17
Nref            2672                      2668
Tmin,Tmax       0.511,0.685               0.506,0.679
Tmin’           0.422

Correction method= # Reported T Limits: Tmin=0.506 Tmax=0.679
AbsCorr = MULTI-SCAN

Data completeness= 0.999          Theta(max)= 26.406

R(reflections)= 0.0385( 1906)     wR2(reflections)= 0.0740( 2668)

S = 1.014                  Npar= 194
```

The following ALERTS were generated. Each ALERT has the format
test-name_ALERT_alert-type_alert-level.
Click on the hyperlinks for more details of the test.

Alert level C

```
PLAT906_ALERT_3_C Large K Value in the Analysis of Variance ......      2.252 Check
```

Alert level G

```
PLAT004_ALERT_5_G Polymeric Structure Found with Maximum Dimension          1 Info
PLAT066_ALERT_1_G Predicted and Reported Tmin&Tmax Range Identical          ? Check
PLAT128_ALERT_4_G Alternate Setting for Input Space Group     C2/c       I2/a Note
PLAT232_ALERT_2_G Hirshfeld Test Diff (M-X)  Cu1      --O3_b     .        7.4 s.u.
PLAT794_ALERT_5_G Tentative Bond Valency for Cu1       (II)      .       2.19 Info
PLAT910_ALERT_3_G Missing # of FCF Reflection(s) Below Theta(Min).          1 Note
PLAT912_ALERT_4_G Missing # of FCF Reflections Above STh/L=  0.600          3 Note
PLAT933_ALERT_2_G Number of OMIT Records in Embedded .res File ...          1 Note
PLAT978_ALERT_2_G Number C-C Bonds with Positive Residual Density.          3 Info
```

```
   0 ALERT level A = Most likely a serious problem - resolve or explain
   0 ALERT level B = A potentially serious problem, consider carefully
   1 ALERT level C = Check. Ensure it is not caused by an omission or oversight
   9 ALERT level G = General information/check it is not something unexpected

   1 ALERT type 1 CIF construction/syntax error, inconsistent or missing data
   3 ALERT type 2 Indicator that the structure model may be wrong or deficient
   2 ALERT type 3 Indicator that the structure quality may be low
   2 ALERT type 4 Improvement, methodology, query or suggestion
   2 ALERT type 5 Informative message, check
```

It is advisable to attempt to resolve as many as possible of the alerts in all categories. Often the minor alerts point to easily fixed oversights, errors and omissions in your CIF or refinement strategy, so attention to these fine details can be worthwhile. In order to resolve some of the more serious problems it may be necessary to carry out additional measurements or structure refinements. However, the purpose of your study may justify the reported deviations and the more serious of these should normally be commented upon in the discussion or experimental section of a paper or in the "special_details" fields of the CIF. checkCIF was carefully designed to identify outliers and unusual parameters, but every test has its limitations and alerts that are not important in a particular case may appear. Conversely, the absence of alerts does not guarantee there are no aspects of the results needing attention. It is up to the individual to critically assess their own results and, if necessary, seek expert advice.

Publication of your CIF in IUCr journals

A basic structural check has been run on your CIF. These basic checks will be run on all CIFs submitted for publication in IUCr journals (*Acta Crystallographica*, *Journal of Applied Crystallography*, *Journal of Synchrotron Radiation*); however, if you intend to submit to *Acta Crystallographica Section C* or *E* or *IUCrData*, you should make sure that full publication checks are run on the final version of your CIF prior to submission.

Publication of your CIF in other journals

Please refer to the *Notes for Authors* of the relevant journal for any special instructions relating to CIF submission.

PLATON version of 19/10/2018; check.def file version of 15/10/2018

Datablock shelx - ellipsoid plot

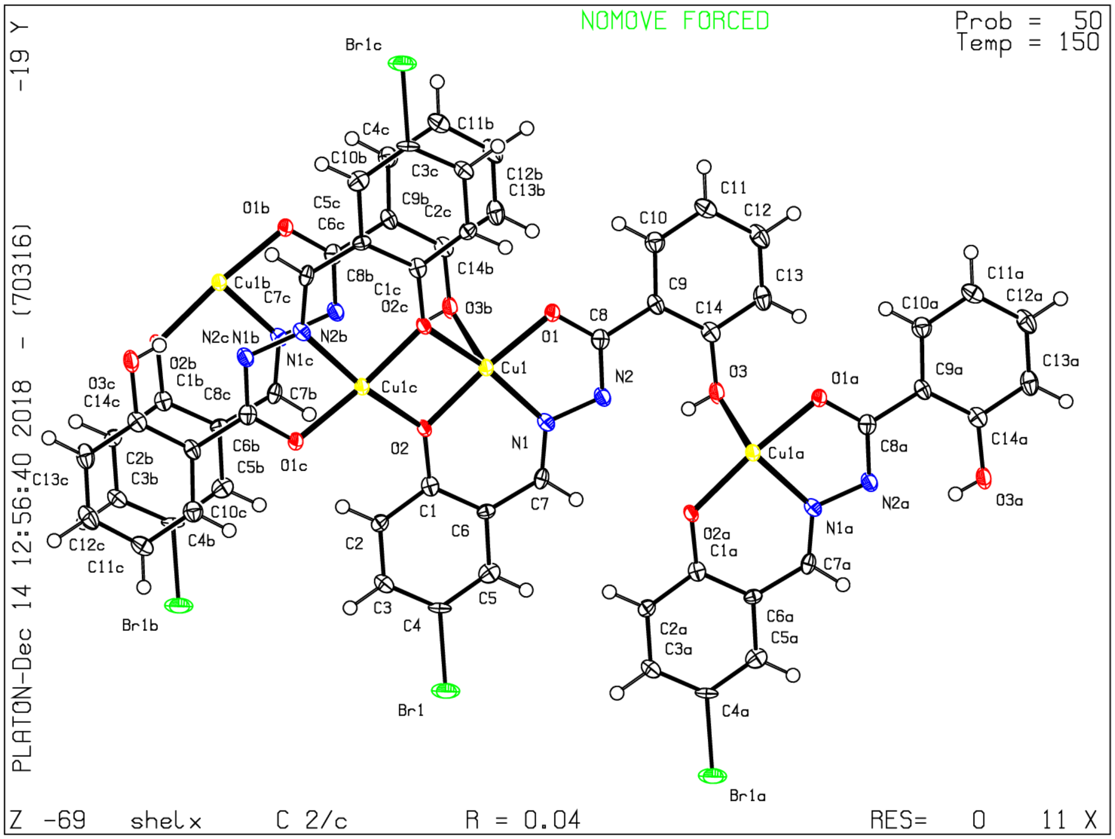

Supplement: Supplementary file 1 [file molecules-24-00047-s001.pdf]
